# Antibody-Drug Conjugates Targeting the Urokinase Receptor (uPAR) as a Possible Treatment of Aggressive Breast Cancer

**DOI:** 10.3390/antib8040054

**Published:** 2019-11-05

**Authors:** Efrat T. Harel, Penelope M. Drake, Robyn M. Barfield, Irene Lui, Shauna Farr-Jones, Laura Van’t Veer, Zev J. Gartner, Evan M. Green, André Luiz Lourenço, Yifan Cheng, Byron C. Hann, David Rabuka, Charles S. Craik

**Affiliations:** 1 Department of Pharmaceutical Chemistry, University of California, San Francisco, CA 94158, USA; efrat.t.harel@gmail.com (E.T.H.); irene.lui@ucsf.edu (I.L.); zev.gartner@ucsf.edu (Z.J.G.); Andre.Lourenco@ucsf.edu (A.L.L.); 2 Catalent Biologics, West, Emeryville, CA 94608, USA; Penelope.drake@catalent.com (P.M.D.); robyn.barfield@catalent.com (R.M.B.); david.rabuka@gmail.com (D.R.); 3 Department of Anesthesia and Perioperative Care, University of California, San Francisco, CA 94110, USA; shauna.farr-jones@ucsf.edu; 4 Laboratory Medicine, Helen Diller Family Comprehensive Cancer Center, University of California, San Francisco, CA 94158, USA; Laura.Vantveer@ucsf.edu; 5 Biophysics Graduate Program and Department of Biochemistry and Biophysics, University of California, San Francisco, CA 94158, USA; green.evan@ucsf.edu; 6 Howard Hughes Medical Institute, University of California San Francisco, and Department of Biochemistry and Biophysics, University of California, San Francisco, CA 94158, USA; ycheng@ucsf.edu; 7 Preclinical Therapeutics Core, UCSF Helen Diller Family Comprehensive Cancer Center, San Francisco, CA 94158, USA; Byron.hann@ucsf.edu

**Keywords:** antibody-drug conjugate (ADC), urokinase receptor (uPAR), targeted therapy, triple-negative breast cancer (TNBC), cleavable linker, non-cleavable linker, site-specific conjugations, monomethyl auristatin E (MMAE), maytansinoid

## Abstract

A promising molecular target for aggressive cancers is the urokinase receptor (uPAR). A fully human, recombinant antibody that binds uPAR to form a stable complex that blocks uPA-uPAR interactions (2G10) and is internalized primarily through endocytosis showed efficacy in a mouse xenograft model of highly aggressive, triple negative breast cancer (TNBC). Antibody-drug conjugates (ADCs) of 2G10 were designed and produced bearing tubulin inhibitor payloads ligated through seven different linkers. Aldehyde tag technology was employed for linking, and either one or two tags were inserted into the antibody heavy chain, to produce site-specifically conjugated ADCs with drug-to-antibody ratios of either two or four. Both cleavable and non-cleavable linkers were combined with two different antimitotic toxins—MMAE (monomethylauristatin E) and maytansine. Nine different 2G10 ADCs were produced and tested for their ability to target uPAR in cell-based assays and a mouse model. The anti-uPAR ADC that resulted in tumor regression comprised an MMAE payload with a cathepsin B cleavable linker, 2G10-RED-244-MMAE. This work demonstrates in vitro activity of the 2G10-RED-244-MMAE in TNBC cell lines and validates uPAR as a therapeutic target for TNBC.

## 1. Introduction

A potential molecular target for aggressive cancers is the plasminogen activation system (PAS), which plays a key role in tissue degradation during cancer invasion. It has been known for over two decades that overexpression of the serine protease urokinase plasminogen activator (uPA) and its receptor uPAR contributes to the aggressive phenotype in a number of cancers such as breast (both invasive and non-invasive), lung, and gastrointestinal tumors and their metastases, and that this expression is not observed in non-diseased tissue [1,2,3,4,5,6]. Several groups have shown that uPAR expression in breast tumor tissue is highly correlated with metastasis, aggressive phenotypes, poor clinical prognoses, low disease-free survival, and tamoxifen resistance [1,4,5,7,8,9,10]. Expression of uPAR has also been reported on stromal cells [9,11]. Thus, the ubiquity of uPAR in aggressive cancer types and their associated tumor microenvironment makes it an attractive molecular target, especially for TNBC where there is a paucity of molecular targets. Others have shown with both knockout studies and extensive work on mouse uPAR that significant toxicities are not seen when mouse uPAR function is blocked [12].

Breast cancer death rates are the second highest, after lung cancer, for women in the U.S. [13]. Breast cancer is a heterogeneous disease comprised of multiple subtypes that respond distinctly to different therapeutic regimens. Triple-negative breast cancer (TNBC) is an aggressive subtype representing 15–20% of invasive breast cancer cases in women, depending on ethnicity [13]. TNBC patients have a high rate of recurrence with a poor prognosis. TNBC most commonly metastasizes to visceral organs, including lung, liver, brain, and bone. Once a primary TNBC tumor has metastasized, death generally follows within two to three years [14]. TNBC tumors lack the estrogen and progesterone receptor (ER/PR), the human epidermal growth factor 2 (HER2), and do not respond well to current therapies. Despite the U.S. Food and Drug Administration (FDA) approval of several new breast cancer drugs, there remain few therapeutic options for TNBC patients [15,16]. Molecular targets in this aggressive subtype are needed as they can be used as both diagnostic probes for disease evaluation and as targeting functionalities to enable disease treatment. 

Based on the importance of uPAR as a molecular target in cancer, a fragment–antigen-binding (Fab) antibody phage display library was used to identify fully-human recombinant anti-uPAR antagonist antibodies [17]. Twelve antibodies were selected using uPAR folded in its native conformation to enable three-dimensional epitope recognition. The IgG1 antibody referred to as 2G10 was chosen for further development due to its uPAR antagonistic properties [17]. The antibody 2G10 forms a stable complex with uPAR, disrupts uPA-uPAR interactions and showed diagnostic and therapeutic potential in vitro and in TNBC models and a metastatic mouse model [17,18,19]. Because the 2G10 Fab was selected from a human phage display library, and expressed as an IgG with the trastuzumab (Herceptin, Genentech) Fc domain, it is potentially amenable for use in therapeutic applications. 2G10 is a high-affinity binder to uPAR (Fab K_D_ = 10 × 10^−9^; IgG K_D_ = 2 × 10^−12^) [18]. Our previous work showed that the 2G10 antibody has attributes of a slow off rate to the antigen (uPAR) and internalization that are required for use as an ADC. Also, its ability to block the localization of uPA to the pericellular membrane of a tumor provides an independent mechanism for controlling tumor cell growth and metastasis. In a mouse model of TNBC, 2G10 IgG at high-doses (30 mg/kg) blocked tumor growth as a monotherapy [18]. We also showed that a ^177^Lu radiolabeled 2G10 virtually eliminated the tumors in an orthotopic xenograft breast cancer model [18]. Based on these promising preclinical results and the practical limitations of using a radiolabeled antibody, 2G10 antibody-drug conjugates (ADCs), were designed and tested. Potent cytotoxic agents were selectively attached to the antibody, to maximize anti-tumor responses while minimizing toxicity and enhance 2G10 therapeutic potential. As an ADC, 2G10 shows the promising attributes of the non-ADC antibody in that it blocks uPA binding, internalizes and is highly specific, presumably due to the site-specific conjugation of the payload to the Fc portion of the antibody at a site(s) distal to the antigen-binding region.

ADCs constitute a class of cancer therapeutics designed to selectively eliminate cancer cells expressing a cell-surface antigen recognized by an antibody. The ADC approach has been clinically validated with five ADCs currently marketed, over 60 ADCs currently in clinical evaluation, and many more in preclinical development [20]. For the effective generation and application of ADCs many characteristics need to be optimized, such as the antibody specificity and the cytotoxic payload selection [21,22,23,24,25]. Important parameters affecting an ADC’s therapeutic window relate to the conjugation chemistry and the functional linker that binds the antibody to the payload. Historically, cytotoxic payloads have been conjugated via chemically-reactive amino acid side chain residues (e.g., on lysines and cysteines) leading to heterogeneous conjugate populations. In contrast, controlled, site-specific conjugation has the potential to overcome heterogeneity and improve the therapeutic window [20,21,22,23,26,27,28,29,30,31,32,33]. In the current study, three ADC elements and their corresponding effects on potency in a TNBC model were compared: (I) the linker being cleavable vs. non-cleavable; (II) the toxin as MMAE vs. maytansine, and; (III) the drug-to-antibody (DAR) ratio as DAR 2 vs. DAR 4. The anti-uPAR 2G10 ADCs presented in this study were prepared using the SMARTag^®^ site-specific bioconjugation technology that incorporates an aldehyde tag conjugated using hydrazino-iso-Pictet-Spengler (HIPS) chemistry [21,26,27,28,32,33]. The aldehyde tag approach enables site-specific modification yielding homogeneous ADCs with defined attachment sites for the toxin. The tag consists of a five amino acid consensus sequence (CxPxR) inserted genetically into the desired location within the antibody. During antibody production, the formylglycine-generating enzyme recognizes the tag sequence and co-translationally converts the cysteine residue to a formylglycine residue containing an aldehyde functional group, which serves as the chemical handle for bio-orthogonal conjugation [34]. The reactive aldehyde can then undergo conjugation to the drug-linker via a hydrazino-iso-Pictet-Spengler (HIPS) ligation resulting in a carbon-carbon bond between the antibody and toxin that leads to a highly-stable ADC both in vitro and in vivo. Recently, a novel SMARTag^®^-based anti-CD22 ADC, TRPH-222, has entered clinical trials (NCT03682796).

The SMARTag technology was used to attach the microtubule-binding anti-mitotic drugs monomethyl auristatin E (MMAE) and maytansine to the 2G10 antibody using seven linkers. The resulting ADCs were tested and validated in vitro and using a TNBC xenograft model. 

## 2. Materials and Methods

### 2.1. Cell Culture

Human breast cancer cell lines MDA-MB-231, HCC1569, SK-Br3, and MCF-7 were purchased from American Type Culture Collection (ATCC, Manassas, VA, USA) and were maintained in their respective recommended media, supplemented with 10% FBS, 100 U/mL penicillin, and 100 mg/mL streptomycin at 37 °C. Normal human mammary epithelial cells (HMEC, Lonza Group AG, Basel, Switzerland) and cultured using the MEGM^TM^ BulletKit^TM^ (Lonza).

### 2.2. Cloning, Expression, and Purification of Aldehyde-Tagged IgG

Antibody 2G10 (lambda light chain) was identified from a phage display library as previously described in Duriseti et al. [17] and the aldehyde-tagged antibody was produced as described in Drake et al. 2014 [35]. The aldehyde tag sequence was inserted into the heavy chain Fc domain using standard molecular biology techniques. Expi-CHO-S cells stably-expressing human FGE were used for the transient production of antibodies. Antibodies were purified from the conditioned medium using Protein A chromatography (MabSelect, GE Healthcare Life Sciences, Pittsburg, PA, USA). Purified antibodies were stored at 4 °C until use.

### 2.3. Bioconjugation, Purification, and HPLC Analytics of the anti-uPAR the Antibody Drug Conjugates

Bioconjugation, purification, and HPLC analysis were performed as previously described [35]. Aldehyde-tagged antibodies (15 mg/mL) were conjugated to HIPS-linker-payloads (8 mol equiv DAR) for 72 h at 37 °C in 20 mM sodium citrate, 50 mM NaCl pH 5.5 and up to 2.5% DMA. Free drug was removed using a 40 kDa Zeba^TM^ spin column (Thermo Fisher Scientific, Waltham, MA, USA) equilibrated with 20 mM sodium citrate, 50 mM NaCl pH 5.5. To determine the DAR of the final product, ADCs were examined by analytical HIC (Cat. #14947 Tosoh Corp., Tokyo, Japan) with mobile phase A: 1.5 M ammonium sulfate, 25 mM sodium phosphate pH 7.0, and mobile phase B: 25% isopropanol, 18.75 mM sodium phosphate pH 7.0. To determine aggregation, samples were analyzed using analytical size exclusion chromatography (SEC; Cat. #08541, Tosoh Corp., Tokyo, Japan) with a mobile phase of 300 mM NaCl, 25 mM sodium phosphate pH 6.8.

### 2.4. Flow Cytometry of Breast Cancer Cell Lines to Determine uPAR levels 

The breast cancer cell lines were cultured in DMEM-H21 supplemented with 10% heat-inactivated FBS. Cells were washed with DPBS and harvested with TrypLE (Gibco^TM^, Thermo Fisher Scientific, Waltham, MA, USA). 1 × 10^6^ cells were incubated with 2 µg/mL goat anti-human uPAR (R&D Systems, Minneapolis, MN, USA) for 60 min at 4 °C, followed by Alexa Fluor 647-labeled anti-goat antibody (Invitrogen, Carlsbad, CA, USA) for another 60 min at 4 °C. Stained samples and controls were assayed on a BD FACSCalibur^TM^ (BD BioSciences, San Jose, CA, USA). All experiments were performed in triplicate.

### 2.5. Western Blotting of the 2G10 Fab

Purified uPAR (5 μg) under reducing (33 mM TCEP (tris(2-carboxyethyl)phosphine) reducing agent) and non-reducing conditions were subjected to electrophoresis on both native and SDS-PAGE and transferred to PVDF membranes. Nonspecific binding was inhibited by incubation in TBST [20 mM Tris-buffered saline (pH 7.5) with 0.1% Tween 20] containing 5% non-fat dry milk for 1 h at room temperature (RT). Gels were stained with InstantBlue™ Coomassie Protein Stain (Expedeon Inc., San Diego, CA, USA) while membranes were primarily incubated with either goat polyclonal anti-uPAR antibodies (AF807 10 µg/mL, R&D systems) or 2G10 (10 µg/mL) expressed on a human IgG scaffold in 5% non-fat dry milk for 1 h, washed three times with TBST for 15 min and incubated at RT for 60 min with horseradish peroxidase (HRP)-conjugated secondary antibodies diluted 1:1000. Afterwards, membranes were washed again three times for 15 min with TBST. The chemiluminescence emitted from Immobilon Forte Western HRP Substrate (Millipore Sigma, Burlington, MA, USA) after treatment with antibody-conjugated HRP and proteins thus labeled were visualized using the ChemiDoc XRS+ Imaging system (BioRad Laboratories, Inc., Hercules, CA, USA).

### 2.6. Electron Microscopy of the 2G10-uPAR Complex

Negative Stain Electron Microscopy: uPAR-Fab complexes were prepared by mixing purified uPAR or Fab and uPAR at a 7:1 molar ratio prior to running size-exclusion chromatography on a s200 column using 20 mM HEPES pH 7.5 and 150 mM NaCl as the running buffer. Fractions corresponding to uPAR alone or Fab-uPAR complexes were diluted to a concentration of 0.025 mg/mL. Negative stain EM grids were prepared following established protocols [36]. Briefly, 2.5 μL of sample was applied to a glow discharged carbon-coated Cu EM grid (Ted Pella Inc., Redding, CA, USA) and stained with 0.75% uranyl formate. The sample was imaged on a T20 microscope (FEI Company, Hillsboro, OR, USA) operated at 200 kV with a nominal magnification of 50,000× using a TemF816 8 K × 8 K CMOS camera (TVIPS GmbH, Gauting, Germany) with a calibrated pixel size of 1.57 Å. All images were further binned by 2 for image processing yielding a pixel size of 3.14 Å. Defocus values were determined using gctf and particles were picked using a Gaussian template with gautomatch [37]. Particle extraction was done with RELION-2 [38] and two-dimensional reference-free classification was done using cryoSPARC [39]. 

### 2.7. Antibody Dye Conjugation and Internalization Assay

Purified 2G10 IgG and Fab fragments of 2G10 anti-human uPAR from primary amine-free stock solutions were diluted to 1 mg/mL in PBS containing 50 mM NaHCO_3_ (pH 8.3), and final antibody concentration was determined by absorption measurement at 280 nm. Alexa Fluor (AF)647 N-hydroxysuccinimide (NHS) ester (A20106, ThermoFisher) or the pH sensitive dye, pHAb (G9841, Promega) were reconstituted according to the manufacturer’s protocol. A 20-fold molar excess of amine reactive dye was added to the antibody solutions and incubated for 1 h at room temperature in the dark followed by dialysis against PBS overnight. The unbound dye was removed by fractionation over a PD-10 desalting column. Internalization was evaluated by flow cytometry (BD FACSCalibur^TM^). MDA-MB-231 cells were incubated with 2G10-AF647 (10 µg/mL at 37 °C) analyzed at 0.5, 1, 2, and 4 h. Internalization of 2G10-AF647 or 2G10-amine-pHAb was also monitored using confocal microscopy. MDA-MB-231 cells were incubated for 1 h at 37 °C with 10 µg of 2G10-AF647 and lysosome dye 50 nM LysoTracker Green DND-26, or with 10 µg 2G10-amine-pHAb. Excess antibody was washed away and cells were imaged on Yokagawa CSU22 spinning disk confocal (Ex/Em: 561/580LP nm). In the same confocal microscopy assay, the effect of the chaperone Receptor-Associated Protein (RAP) and endocytosis inhibitors on 2G10-AF647 internalization was monitored. Inhibitors of clathrin-mediated endocytosis were tested at low temperature, by performing the entire experiment at 4 °C; using PitStop2 at a concentration of 25 μM; or Dynasore at a concentration of 100 μM for 30 min prior and during to a one-hour incubation with 2G10-AF647.

### 2.8. In Vitro Cytotoxicity Assays

MDA-MB-231 cells were plated in 96-well plates (Costar 3610) at a density of 5 × 10^4^ cells/well in 100 μL of growth media and allowed to rest for 24 h. Serial dilution of ADC or 2G10 samples was performed in DMEM at 6× the final concentration and 20 μL was added to the cells. ADC concentration was determined using A280 nm with an extinction coefficient of 1.6 for ADCs, 1.48 for 2G10, and MWt of 150 kDa. After incubation at 37 °C with 5% CO_2_ for 5 days, viability was measured using a Promega CellTiterGlo kit following the manufacturer’s protocol. Data was collected on a SpectraMax M5 plate reader using luminescence settings. Data was normalized to controls on each plate.

### 2.9. Xenograft Assays

All animal studies were conducted under an animal use protocol approved by the University of California, San Francisco animal care and use committee approved on 7 May 2018. Female C.B-17 SCID mice were inoculated subcutaneously with 1 × 10^7^ MDA-MB-231 cells in 50% Matrigel. When the tumors reached an average of 100 mm^3^, animals were randomized into groups of 10 or 4 mice and were dosed as described. The animals were given four doses of 5 or 10-mg/kg of ADC, 2G10 antibody (untagged), or vehicle alone. The animals were monitored twice weekly for body weight and tumor size. Animals were also monitored closely for signs of discomfort or pain. Animals were checked once per week prior to treatment and three times weekly during treatment for signs of distress including ruffled fur, weight loss approximating 15% body weight, diarrhea, lack of appetite, or hunched in the cage corner. Tumor volume was estimated according to the formula:

$$\mathrm{tumor}\;\mathrm{volume}\;\left({\mathrm{mm}}^{3}\right)=\frac{w^{2}\;l}{2}$$

where *w* is tumor width and *l* is tumor length. Animals were euthanized at the end of the study, or when tumors reached 2 cm^3^.

### 2.10. Statistical Analysis

All in vitro experiments were performed in triplicate. Statistical analysis was performed using GraphPad Prism software (VERSION 6.0, Graphpad Software Inc., San Diego, CA, USA). For determination of statistical significance, ANOVA was performed, followed by the Holm-Šídák correction for multiple comparisons for tumor volume measurements. 

## 3. Results

**Figure 1:**
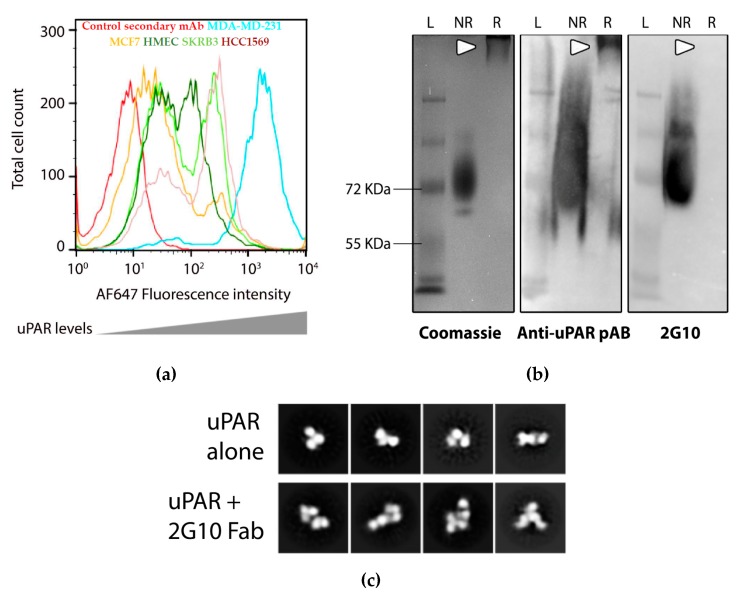
2G10 binding to recombinant and cell surface urokinase receptor (uPAR). (**a**) Cell surface levels of uPAR were measured using a goat derived anti-human uPAR antibody that was detected with a secondary anti-goat antibody by immunofluorescent staining and flow cytometry. uPAR levels are represented detected on the surface of TNBC cell lines MDA-MB-231 (cyan), MCF7 (ER+ /PR+, orange), SKRB3 (ER-/PR-/HER2+, light green), HCC1569 (ER-/PR-/HER2+, brown), HMEC (dark green). All cell lines were stained with an anti-goat secondary antibody. The non-specific binding of the secondary antibody control is shown in red. Representative curves from three runs are shown. Standard deviation of peaks are: MDA-MB-231, ±170; HCC1569, ±42.8; SKRB3, ±35.6; MCF7, ±17.9; HMEC, ±15.6. (**b**) Western blot of reduced and non-reduced uPAR following SDS-PAGE. White arrow indicates uPAR location on gel. Left: Coomassie-stained, center: blotted with polyclonal anti-uPAR, right: gels were blotted with 2G10 Fab on a rabbit IgG scaffold and probed with a secondary anti-rabbit conjugated to horseradish peroxidase (HRP). **L** indicates sizing ladder, **NR** indicates non-reducing conditions, **R** indicates reducing conditions. (**c**) Representative nsEM 2D class averages of monomeric uPAR and uPAR-2G10 Fab complexes. Box size for the 2D class averages is 201 Å.

**Figure 2:**
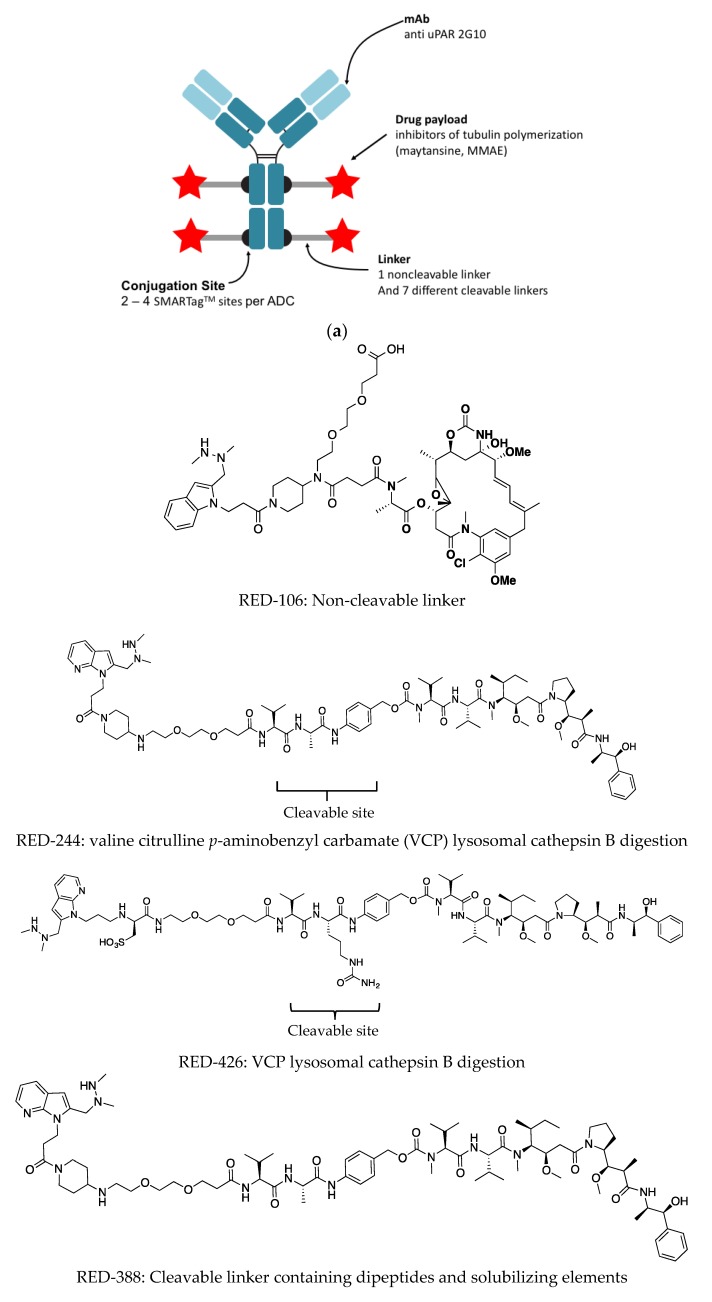

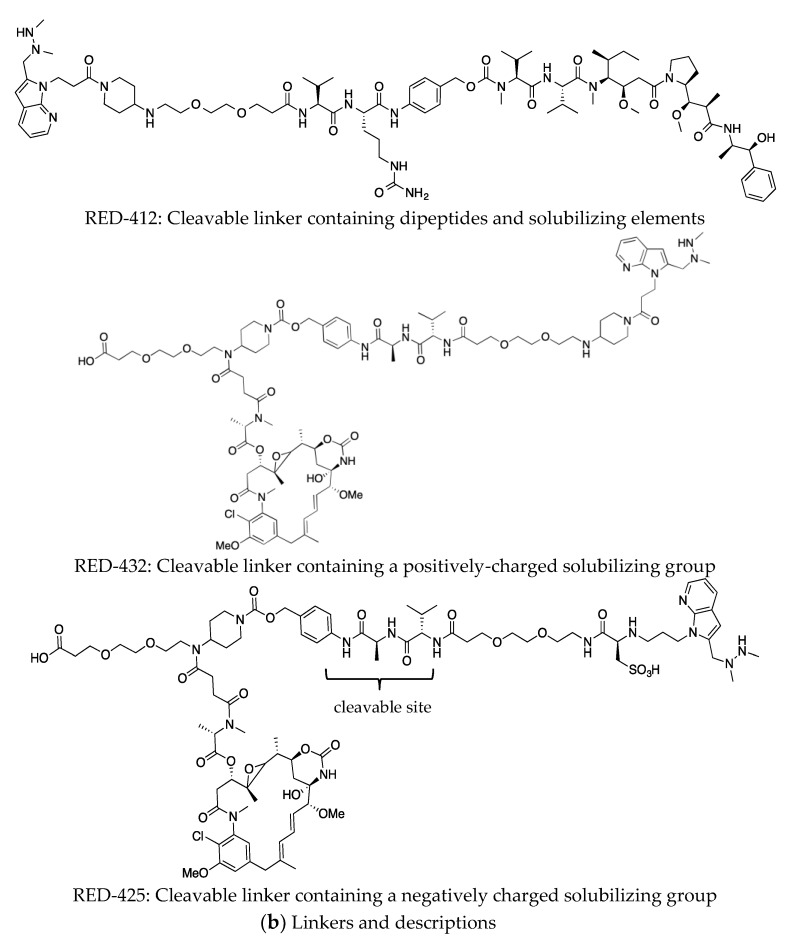
Design of antibody-drug conjugates. (**a**) Schematic of the components of site-specifically modified 2G10 anti-uPAR ADC. Antibodies carrying aldehyde moieties (DAR of 2 or 4) are reacted with a Hydrazino-iso-Pictet-Spengler (HIPS) linker/payload to generate a site-specifically conjugated ADC. Inhibitors of tubulin polymerization (maytansine, monomethyl auristatin E, MMAE) linked to cleavable or non-cleavable linkers. (**b**) The chemical composition of the linkers. Only RED-106 is non-cleavable.

Flow cytometry of various cell lines indicated that MDA-MB-231 cells display the highest level of uPAR (Figure 1a), and have been previously shown to have higher uPAR RNA level expression compared to other breast cancer cell lines [18]. After MDA-MB-231, uPAR expression was observed on HCC1569 and SKRB3 cells, both of which are ER-/PR-/HER2+. The non-tumorigenic human mammary epithelial cells showed low uPAR expression, as did the ER+ /PR+ cell line, MCF7 (Figure 1a). The binding of the human recombinant anti-uPAR 2G10 to uPAR was previously investigated [17], and we reported that 2G10 Fab and IgG binds uPAR using surface plasmon resonance with K_D_ values of 10 × 10^−9^ and 2 × 10^−12^ mol/L, respectively [18]. 2G10 competes for binding with uPA, but it was not known whether the antibody binds uPAR in the uPA-binding site or in a distant site that induces a conformational change that prevents uPA binding. 2G10 does not recognize the reduced, unfolded uPAR as shown using Western blotting (See SDS-PAGE gels, Figure 1b). Denatured, reduced uPAR did not migrate into the gel. Binding of 2G10 Fab to uPAR was further characterized by negative stain electron microscopy (nsEM, Figure 1c). The three-domain architecture of uPAR alone is observed in 2D class averages and the 2G10 Fab can be identified by the canonical Fab shape observed in nsEM [40]. Owing to the resolution limits of nsEM it is not possible to define the binding epitope from the nsEM 2D class averages. In the present study, we designed and tested nine ADCs incorporating our 2G10 anti-uPAR antibody (Figure 2). The anti-uPAR ADCs were generated using aldehyde tag technology coupled with HIPS chemistry to achieve site-specific bioconjugation [35,41,42,43,44]. 

The antibody was produced and purified using conventional means, followed by direct conjugation to an aldehyde-reactive payload as previously described by Drake et al. 2014 [34,35]. The DAR is defined by the number of aldehyde tags incorporated per antibody. Either one or two aldehyde tags were incorporated into the IgG heavy chain (Fc portion), resulting in two or four conjugation sites per antibody (Figure 2a, Table 1). A HIPS-functionalized cleavable or non-cleavable linker covalently linked the drug to the antibody. The choice of a linker and whether it is cleavable or not, can impact the biophysical and functional properties of the ADC. Seven different linkers exhibiting various properties of interest (Figure 2b) were compared. The non-cleavable linker, RED-106, was designed to persist through antibody degradation in the lysosome (Figure 2b) [27]. 

**Figure 3:**
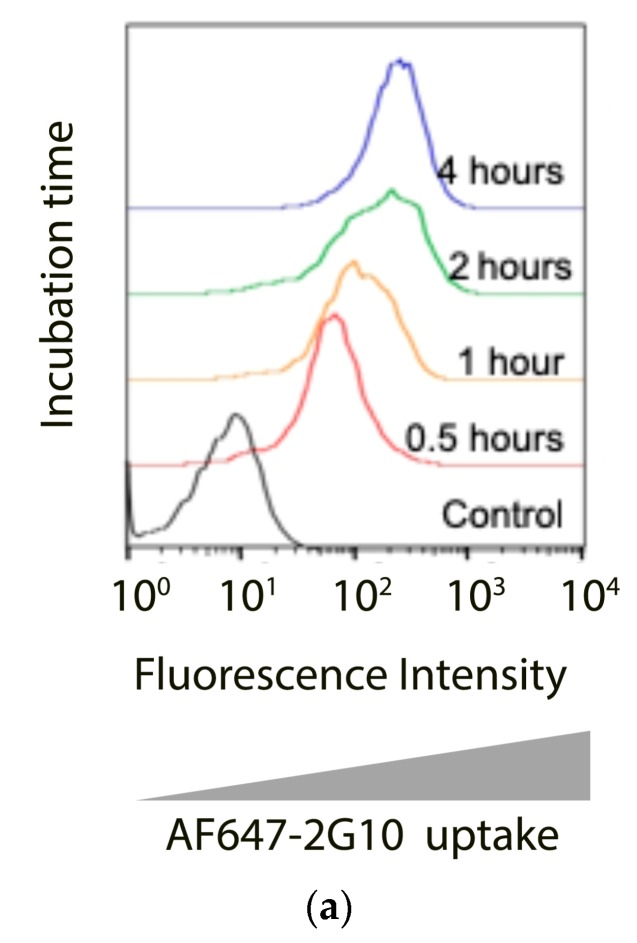

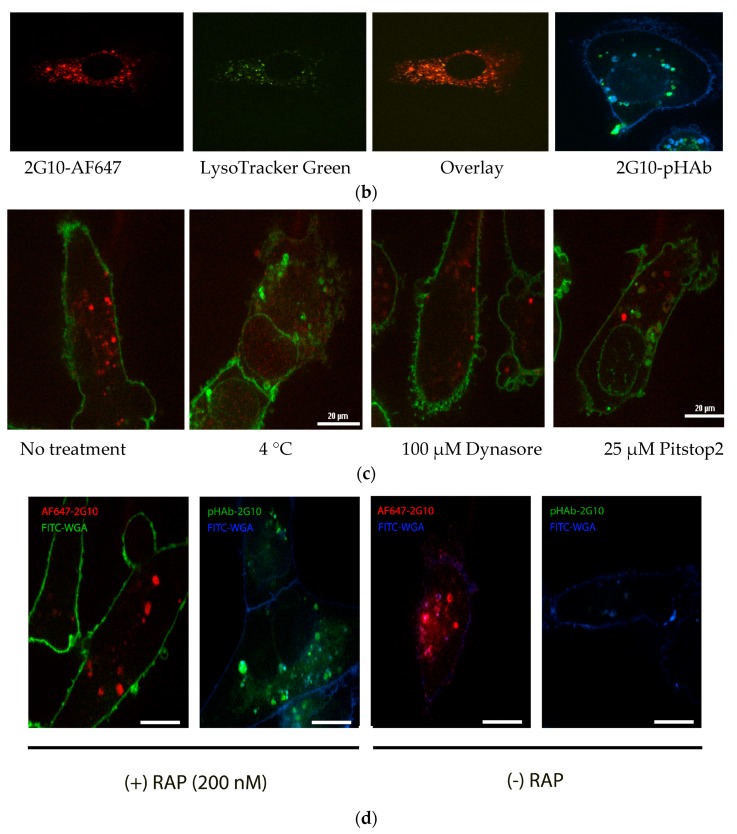
2G10 internalization by MDA-MB-231 cells. (**a**) Flow cytometry analysis of 10 µg/mL 2G10 internalizing at 37 °C into MDA-MB-231 cells at various timepoints. Positive binding is indicated by a rightward shift in fluorescence intensity of histogram. (**b**) Internalization of 2G10-AF647 or 2G10-amine-pHAb monitored using confocal microscopy. MDA-MB-231 cells were incubated for 1 h at 37 °C with 10 µg of 2G10 IgG conjugated to Alexa Fluor 647 (red, 2G10-AF647) and lysosome dye LysoTracker Green DND-26 (green), or with 10 µg 2G10-amine-pHAb. Excess antibody was washed away and cells were imaged on Yokagawa CSU22 spinning disk confocal (Ex/Em: 561/580 LP nm). The top panel shows the confocal analysis showing 2G10-AF647 (red) localized to the lysosome, which is stained with a lysosome-specific green dye. The overlay image showed high colocalization of 2G10-AF647 into the lysosome (yellow). (**c**) The effect of endocytosis inhibitors on 2G10-AF647 internalization was monitored using confocal microscopy. MDA-MB-231 cells were incubated for 1 h at 37 °C with 10 µg of 2G10-AF247. Cell membranes were visualized with wheat germ agglutinin (WGA)-AF488 (green) which binds to cell membrane. Excess antibody was removed and replaced with pH 8.0 buffers and imaged on Yokagawa CSU22 spinning disk confocal (Ex/Em: 561/580 LP nm). (**d**) Antibody internalization to MDA-MB-231 cells in the presence or absence of endocytosis inhibitor RAP (200 nM). 2G10-AF647 (red) or 2G10-pHAb is used for antibody tracking. WGA-AF488 (green) or WGA-AF350 (blue) binds to cell membrane.

Once bound to the target cell-surface antigen, the conjugate must be processed to release an active form of the drug, which can reach its intracellular target (Figure 3). To assess the mechanism of cellular uptake of 2G10, MDA-MB-231 cells were pre-incubated with 2G10 conjugated to Alexa Fluor 647 (2G10-AF647, 10 µg/mL) and incubated with cells for 0.5–4 h at 37 °C. Then, membrane-bound 2G10-AF647 was released by a brief acid wash and the internalized 2G10-AF647 was measured by flow cytometry. 2G10-AF647 accumulated over four hours (Figure 3a). Previous work showing an isotype control antibody, incubated at 4 °C followed by acid wash is not internalized, was previously published [19] and is not shown here.

Immunofluorescence microscopy was used to localize the 2G10 within MDA-MB-231 cells. We had previously shown that 2G10 does not internalize to MCF7 cells [19]. Lysosomes, visualized using LysoTracker (green signal) were distinct and punctate. MDA-MB-231 incubated with LysoTracker and 2G10-AF647 (red signal), 10 µg/mL, 1 h at 37 °C), and resulted in a yellow signal indicating colocalization of the 2G10-AF647 and the lysosomes. This image showed that most of the ADCs end up in the lysosome (Figure 3b). Conjugation of 2G10 to a fluorophore with very low fluorescence at pH > 7 (pHAb) also shows migration of 2G10 to the lysosome (Figure 3b). Inhibitors of clathrin-mediated endocytosis reduced the internalization of 2G10. Endocytosis inhibitors tested were: low temperature (performing the entire experiment at 4 °C); clathrin inhibitor PitStop^®^ 2 (25 μM); or dynamin inhibitor Dynasore (100 μM) for 30 min prior to a one-hour incubation with 2G10-AF647 (Figure 3c). Low temperature inhibits multiple internalization mechanisms and was able to prevent the internalization of 2G10-AF647, whereas the endocytosis blockers, Dynasore and Pitstop 2 reduced but did not stop internalization. The uPAR-specific endocytosis inhibitor RAP (200 nM) did not affect antibody internalization to MDA-MB-231 (Figure 3d).

**Figure 4:**
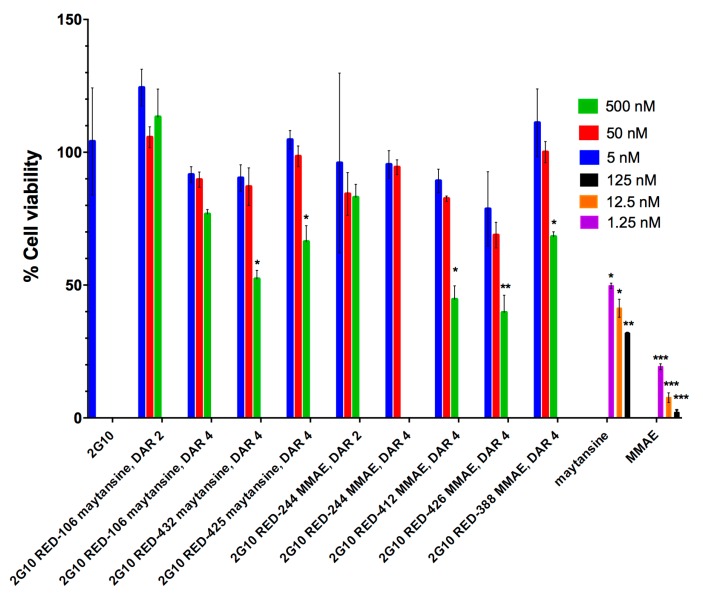
Effect of anti-uPAR 2G10 ADCs, IgG 2G10 alone, and free drug on cell viability. MDA-MB-231 cells (0.5 × 10^4^ cells/well) were seeded in 96-well plates (Costar 3610) at the density and incubated for 120 h (5 days) with of ADCs or IgG. After 5 days of drug treatment at 37 °C with 5% CO_2_, cell viability was measured using the Promega CellTiterGlo kit following the manufacturer’s protocol. Data was collected on a SpectraMax M5 plate reader using luminescence settings. Vehicle-treated cells were set to 100% viability and the luminescence blank was set to 0%. MMAE and maytansine are shown as positive controls. All experiments were performed in triplicate, error bars indicate standard deviation. Asterisks indicate significance level compared to vehicle-treated. Multiplicity-adjusted *p* values from the Holm-Šidák method. * *p* < 0.05; ** *p* < 0.01; *** *p* < 0.001.

Given the finding of internalization of the various ADCs dependent upon uPAR, we evaluated viability of MDA-MB-231 cells to see if internalization would result in cell death and to compare relative cytotoxicity of the various ADCs. Nine DAR 4 2G10-based ADCs bearing different linkers and conjugated to either maytansine or MMAE were compared for their cell-killing ability as compared to the antibody or the payloads alone (1.25–125 nM). All ADCs showed a reduction of cell viability of 17–60% at the highest dose as compared to the vehicle-treated cells (Figure 4). The greatest potency was observed with 2G10-RED-426-MMAE and 2G10-RED-412-MMAE. These two ADCs were significantly more potent than the most potent maytansine-based ADC, (2G10-RED-432-maytansine). Based on this cell viability screen, we selected a set of ADC constructs for further evaluation.

**Figure 5:**
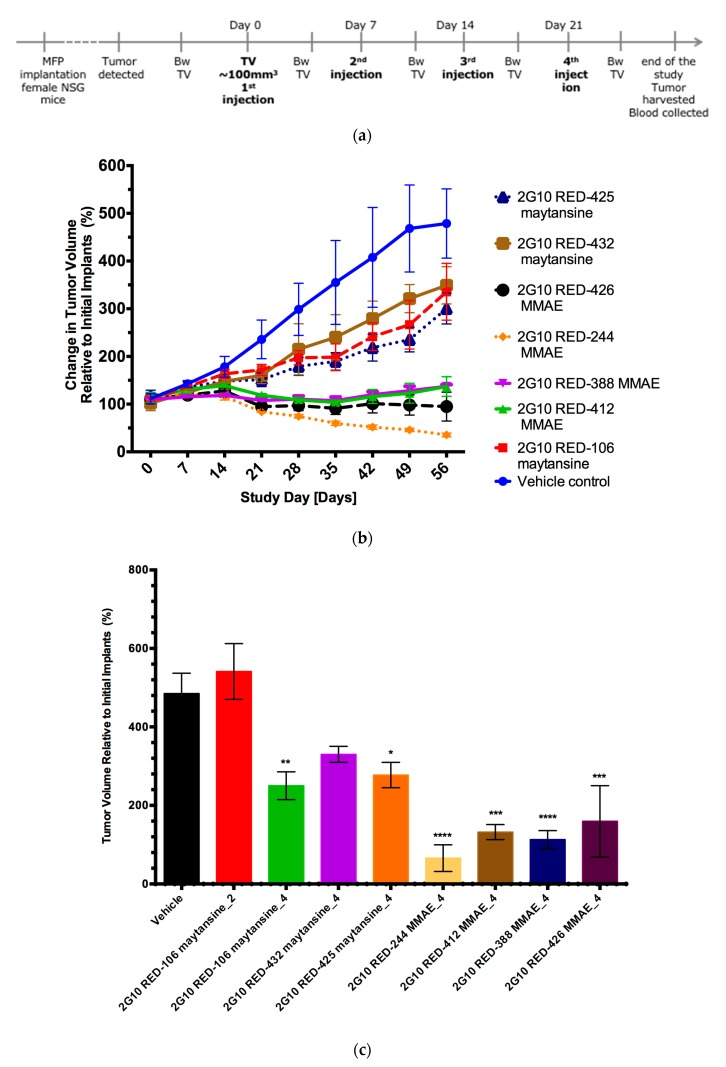

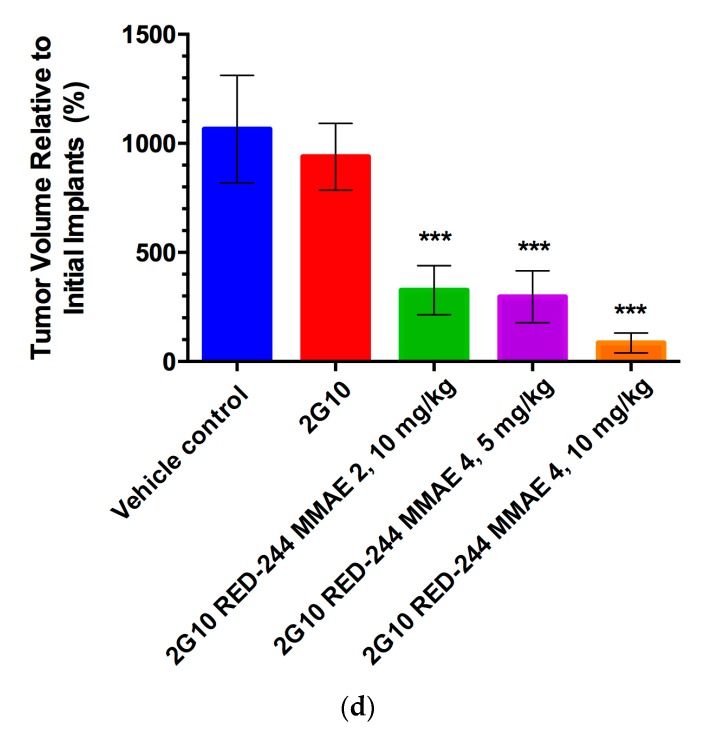
Effect of anti-uPAR 2G10 ADCs on tumor growth (**a**) Schematic of the in vivo experiment to evaluate seven SMARTag based anti-uPAR 2G10 ADCs. Asterisks indicates time of injection. (**b**) Comparison of tumor growth with various 2G10 ADCs coupled with maytansine and MMAE. Linker RED106 is non-cleavable, other are cleavable. (*n* = 4, Error bars indicate SEM) (**c**) Summary of percent tumor volume (Error bars indicate standard deviation) relative to the initial volume (100%, 100 mm^3^) at endpoint for various treatments (all at 10 mg/kg dose). (**d**) 2G10-RED-244-MMAE dose–response (*n* = 10, Error bars indicate standard deviation). For (**c**,**d**) Asterisks indicate significance level comparted to vehicle-treated. Multiplicity-adjusted *p* values from the Holm-Šidák method. * *p* < 0.05; ** *p* < 0.01; *** *p* < 0.001; **** *p* < 0.0001.

To compare the in vivo anti-tumor efficacy of the various ADC constructs, we used MDA-MB-231 xenografts in mice. Animals with tumors of approximately 100 mm^3^ were treated once a week for four weeks with a 10 mg/kg dose of a variety of DAR 4 ADCs or 2G10 antibody alone as described in Figure 5a. 

Nine 2G10 ADCs conjugated as DAR 4 variants bearing MMAE or maytansine were tested. The tumor growth rate in this xenograft model (Figure 5b) was consistent with our previous experience, and that reported by others [45,46] i.e., doubling of tumor volume in approximately 21 days. Specifically, maytansine conjugates reduced final tumor volumes by 1.4- to 1.6-fold compared to the vehicle-treated group. MMAE conjugates final tumor volume was 3.5 to 14-fold less than the vehicle-treated group. The 2G10-RED-244-MMAE ADC showed the greatest efficacy, leading to reduction in tumor volume of one third of the initial volume (Figure 5b). One animal in the vehicle control group died between day 49 and day 56. There was no evidence of toxicity due to ADC treatment based on the absence of clinical observations indicating pain or distress, or from changes in body weight relative to the control group (Figure S1a). 

Reduction in tumor volume relative to vehicle-treated for all tested constructs with statistical parameters is shown in Figure 5c. Based on these results, we reject the null hypothesis of no difference for the four MMAE conjugates tested and for 2G10 RED-106 maytansine, DAR 4 and 2G10 RED-435 maytansine. In particular, 2G10-244-MMAE DAR 4 treatment resulted in tumor volume below the initial volume 65.8 ± 32 mm^3^. 

The best performing ADCs from the first xenograft study were selected and effect of the DAR on ADC anti-tumor efficacy was evaluated. We constructed ADCs with the RED-244 linker carrying the MMAE payload as either DAR 2 or DAR 4 variants and injected either 10 mg/kg (DAR 2) or 5 mg/kg (DAR 4) (Figure 5d). One mouse in the vehicle control group (*n* = 10) died between days 21 and 28, and one mouse in the 2G10_244_MMAE DAR 4, 10 mg/kg group (*n* = 10) died between day 14 and 21 for reasons that were determined by veterinary staff not to be drug-related. There was no significant difference in efficacy observed when the total amount of delivered drug was equal, i.e., 2G10-RED-244-MMAE DAR 4 administered at 5 mg/kg vs. 2G10-RED-244-MMAE DAR 2 at 10 mg/kg (Figure 5d), as would be expected since the total amount of MMAE is the same for these two. When the MMAE dose was increased, i.e., 2G10-RED-244-MMAE DAR 4 at 10 mg/kg improved (compared to 5mg/kg) efficacy was observed, demonstrating a dose–response to ADC treatment. At the lower doses, tumor shrinkage was 3.3–3.6 fold that of the vehicle-treated group (Figure 5d). The greatest response was seen with 2G10-RED-244-MMAE DAR 4 at a dose of 10 mg/kg, which resulted in approximately 12.5-fold shrinkage in tumor volume compared to the vehicle-treated group. There was no evidence of toxicity based on body weight of mice (Figure S1) or clinical observations. 

## 4. Discussion

uPAR is a glycolipid-anchored receptor that brings the serine protease urokinase plasminogen activator to the cell surface to induce peri-cellular proteolysis and engage integrins for cell migration—these activities are opportunities for diagnosis and intervention for cancer treatment. Overexpression of uPAR has been found in several cancers and is common to breast cancer with aggressive phenotypes. This study shows the impact of targeting epithelial expression of uPAR in MDA-MB-231 cells in a xenograft mouse model since it is the most referenced cell line for TNBC, a focus of the study. To determine if it was possible to use MDA-MB-231 cells as in vitro models for uPAR targeting studies, uPAR was measured in MDA-MB-231 cells and in HMEC. We had previously shown that uPAR mRNA expression is elevated in the MDA-MB-231 cell line compared to HMEC [18]. The uPAR protein levels were ~30-fold higher in MDA-MB-231 compared to the HMEC control. In addition, accessibility of uPAR on the cell surface and its overexpression that is associated with an aggressive phenotype makes uPAR a promising molecular target.

Since 2G10 is specific to human uPAR, this model does not directly assess the potential toxicity that could arise if anti-uPAR ADC bound normal mouse cells. However, others have shown with both knockout studies and extensive work on mouse uPAR that significant toxicities are not seen when mouse uPAR function is blocked [12].

A caveat of the current study is that uPAR is expressed on tumor stromal tissue, in addition to breast epithelial cells and this xenograft model does not take stromal expression into account. However, it is worth considering our results in light of recent studies showing that targeting CXCL12 that is expressed in tumor stroma synergizes with anti-PD-L1 immunotherapy [47]. Similarly, a pegylated form of hyaluronidase that can ablate physical barriers in the tumor stroma to improve treatment of pancreatic ductal adenocarcinoma [48]. These studies show that therapeutics developed to target the tumor microenvironment may be effective for malignancies that lack a molecular target on tumor cells such as TNBC.

Advances in antibody engineering, identification of highly cytotoxic molecules, and the generation of linkers with increased stability in circulation have all contributed to the development of the many ADCs that are currently in clinical trials. The aim of the current study is to explore the compatibility of the uPAR target with 2G10 ADCs comprising various linker designs with respect to efficacy against a TNBC model. Despite initial setbacks, efforts to develop ADCs have advanced rapidly in recent years, as clinical trial results coupled with preclinical studies have led to a better understanding of the factors leading to success and the key ADC design characteristics. These factors include: choice of target antigen, conjugation chemistry, DAR, linker design, and cytotoxic payload (Figure 2a). All these parameters must be optimized to create a targeted therapy with improved efficacy and tolerability. The toxins tested in this study were maytansine and MMAE, antimitotic tubulin-targeting drugs that bind to and inhibit tubulin polymerization. Both payloads are components of FDA-approved ADCs [49,50]. By incorporating site-specific and stable conjugation of these payloads to the 2G10 antibody we were able to precisely control the DAR, linker location, and avoid linker instability. 

In many ADCs the toxin is covalently conjugated to the antibody via an amino acid residue side chain, commonly lysine. Random lysine conjugation processes produce heterogeneous mixtures of conjugated species with variable DAR that could impact solubility, stability, and PK of the ADC. Furthermore, having a heterogeneous mixture complicates characterization and product consistency, which could reduce reproducibility. In the current study, aldehyde-specific bio-conjugation was used to enable site-specific modification of an anti-uPAR antibody. This technology resulted in homogeneous ADCs with defined attachment sites for the toxin. 

Once bound to the cell-surface antigen, the conjugate must be processed to release an active form of the drug, which can then encounter its intracellular target. Figure 3a is a schematic of the two potential mechanisms of the intracellular trafficking of anti-uPAR antibodies: (I) the left panel illustrates the mechanism for endocytic clearance of uPAR; (II) the right panel illustrates the macro-pinocytic internalization mechanism coupled with recycling to the cell surface. In the first mechanism, the binding of the ADC to uPAR induces endocytosis in the same manner as the endogenous complex with uPA and the plasminogen activator inhibitor (PAI). In this mechanism, the ADC bound to uPAR proceeds to the lysosome or recycles back to the cell surface. In the lysosome, linker and antibody will be cleaved releasing the cytotoxic payload, which subsequently exits from the lysosomal compartment to find its target in the cytosol, eventually leading to cell death. The second mechanism of the intracellular trafficking pathway of uPAR has been reported by Cortese, et al. where, a macropinocytic mechanism is coupled to rapid recycling to the cell surface [51]. It was previously reported that ^111^In-2G10 internalization via a uPAR-dependent, endocytosis mechanism in MDA-MB-231 cells [19] and anticipated that the 2G10 ADCs would internalize via the same mechanism. To determine the mechanism of cellular uptake, the cells were pre-incubated for 1 h with inhibitors of the endocytosis mechanism prior to the addition of the antibody and subsequent evaluation by fluorescence microscopy (Figure 3c). As shown in Figure 3d, in the presence of RAP (Receptor-Associated Protein), which inhibits uPA:PAI-1 endocytosis, 2G10-AF647 internalization is reduced. Suggesting that RAP did not prevent constitutive internalization of uPAR in MDA-MB-231. This result indicated that the main internalization mechanism is endocytosis rather than pinocytosis. 2G10-AF647 was internalized by MDA-MB-231 cells and was detected in the lysosome.

We tested two different cytotoxic agents. Many cytotoxic agents have been incorporated into ADCs that have failed in clinical trials, possibly because cytotoxics are known to kill rapidly dividing cells, but not the slowly dividing cells that seed some tumors. Furthermore, ADCs may not penetrate tumors to sufficient depth. It has been reported that tubulin inhibitors will only attack tumor cells when they are in a mitotic state [25]. With the goal of improving efficacy and the therapeutic index of ADCs, we have compared two highly potent inhibitors of the assembly of microtubules maytansine and monomethyl auristatin E (MMAE). Both toxins are FDA-approved cytotoxics for ADCs, as exemplified by ado-trastuzumab emtansine (Kadcyla®, maytansine) and brentuxzimab vedotin (MMAE) [49,50]. In vitro, both MMAE and maytansine ADCs induced cell killing at 500 nM without clear superiority of one over the other (Figure 4). However, in vivo MMAE showed higher efficacy than maytansine (Figure 5c) in our mouse studies. Both maytansine and MMAE are inhibitors for tubulin polymerization. The reason for superiority of MMAE in vivo is unclear but could be target-specific (e.g., uPAR) or indication-specific (e.g., TNBC). Interestingly, more ADCs armed with auristatin are currently in clinical trials for TNBC than maytansine ADCs. Out of the six ADCs in clinical trials currently for TNBC, three carry auristatin (Glembatumumab Vedotin (NCT01997333), and PTK7-ADC (NCT03243331), and PF-06647020 (NCT02222922) and one carries a maytansinoid, Anetumab maytansine (NCT02485119).

The next parameter evaluated was the linker that attaches the cytotoxic agent to the antibody. The linker must remain stable in circulation and be selectively released when the drug is intracellular at the target tumor. There are two forms of linkers: cleavable (e.g., via enzymatic cleavage) or non-cleavable. We compared seven different linkers, and one non-cleavable (RED-106). The different cleavable linkers contained dipeptide motifs targeted by cathepsin B and other proteases. The efficacy of linkers containing Val-Cit or Val-Ala sequences, and the effects of solubilizing motifs including the use of positively- and negatively-charged groups (Figure 2) were compared. The Val-Cit cleavable linkers are designed to be cleaved by cathepsin B. Caculitan et al. [52] found that that knockout of cathepsin B did not affect the activity of Val-Cit linker based ADCs in vitro (BT-474M1, SK-BR-3, BJAB, KPL-4, WSUDLCL2 and, GNE-293T) and that proteases other than cathepsin B can also cleave the Val-Cit linker with varying degrees of efficiency. Our studies showed the Val-Cit cleavable linker RED-244 conjugating 2G10 the MMAE, gave the highest effect in vivo, suggesting that cell-based assays though useful as an initial screen for activity, are not predictive of in vivo efficacy.

Our analyses evaluated various determinants of potency of ADCs directed to uPAR. The cytotoxic component was found to be the most important factor, with MMAE being superior to maytansine in the uPAR overexpressing MDA-MB-231 xenograft model. Comparing 2G10 conjugated to maytansine (DAR 4) with either cleavable linker (RED-425 and RED-432) or not cleavable (RED-106), we found ADCs with cleavable linkers were not more potent than the non-cleavable linker. We have also compared the DAR and found no difference between DAR of two and four when the total payload dose was held constant. The maximal dose of ADC 2G10-RED-244–MMAE we gave was 10 mg/kg. The difference in tumor volume observed at doses 5 and 10 mg/kg was not statistically significant (Figure 5d). We previously reported that a high-dose (30 mg/kg) of 2G10 in MDA-MB-231 xenograft mice arrested tumor growth [18]. In the current study we show that coupling MMAE to 2G10 with DAR 4 can arrest tumor growth with a third of that dose.

The maximum tolerated dose of MMAE after a single intravenous dose has been reported to be 450 μg/kg in mice [25]. We administered 22-fold higher dose (10 mg/kg), without observing any reduction in mouse body weight or signs of pain or distress, suggesting our ADC is likely to be relatively safe. (Note that the molecular weights of the different ADCs are within 2% of each other.) However, as stated above, a caveat of interpreting the safety of 2G10 in mice is that this antibody does not bind to mouse uPAR, thus effects of the antibody-mediated via the mouse uPAR are not expected.

The basis of our ADC approach is that uPAR is expressed at low basal levels in non-cancerous healthy human tissue [53,54] and at higher levels in tumors. Expression of uPAR in the vascular endothelium induced by VEGF [55] suggests that uPAR targeted antibodies will localize preferentially to tumor vascular endothelium. Supporting this concept is the finding that AE105 [56], a peptide that binds uPAR with high affinity, is currently in clinical trials (NCT02960724) as a cancer imaging agent. AE105 shows specific decoration of the tumors with no specific accumulation in the vascular endothelium [55]. This provides further encouragement that the activity of the ADC will be localized at tumors.

## 5. Conclusions

In summary, 2G10, an anti-uPAR antibody coupled to microtubule inhibitors exhibited highly effective and selective cancer cell killing in xenograft mouse models of TNBC, validating uPAR as a therapeutic target. The ADC molecule 2G10-RED-244-MMAE induced tumor regression and, as it is based on a fully human antibody scaffold, is a promising candidate for further development as a therapeutic for aggressive cancers, especially TNBC.

## 6. Patents

Patent WO2019113248A1, “Anti-uPAR antibody-drug conjugates and methods of use thereof” inventors Charles Craik, Efrat Harel, David Rabuka, Penelope Drake, Jesse McFarland covers the described antibody constructs.

## Figures and Tables

**Table 1:** Antibody-drug conjugate (ADC) characteristics.

ADC Name	Linker	Payload	SMARTag Site	DAR
2G10 RED-106_maytansine_2	RED-106 (non-cleavable)	maytansine	Heavy chain C-term	1.98
2G10 RED-106_maytansine_4	RED-106	maytansine	Heavy chain CH1/C-term	3.74
2G10 RED-425_maytansine_4	RED-425	maytansine	Heavy chain CH1/C-term	3.46
2G10 RED-432_maytansine_4	RED-432	maytansine	Heavy chain CH1/C-term	3.2
2G10 RED 244 MMAE_2	RED-244	MMAE	Heavy chain C-term	1.9
2G10 RED-244_MMAE_4	RED-244	MMAE	Heavy chain CH1/C-term	3.59
2G10 RED-412_MMAE_4	RED-412	MMAE	Heavy chain CH1/C-term	3.54
2G10 RED-388_MMAE_4	RED-388	MMAE	Heavy chain CH1/C-term	3.54
2G10 RED-426 MMAE_4	RED-426	MMAE	Heavy chain CH1/C-term	3.52

## Supplementary Materials

The following are available online at https://www.mdpi.com/2073-4468/8/4/54/s1, Figure S1: Mouse body weights with various ADC treatments.
