# Supplementary material for: Antibody-Drug Conjugates Targeting the Urokinase Receptor (uPAR) as a Possible Treatment of Aggressive Breast Cancer
Source: Antibodies (Basel). 2019 Nov 5;8(4):54. doi: 10.3390/antib8040054 (PMC6963874; doi:10.3390/antib8040054)
Supplement: Supplementary file 1 [file antibodies-08-00054-s001.pdf]

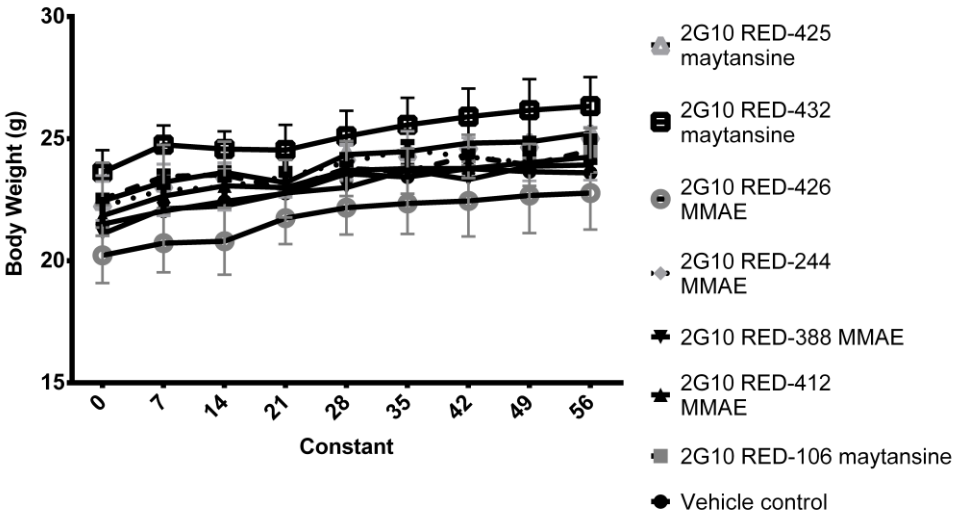

(a)

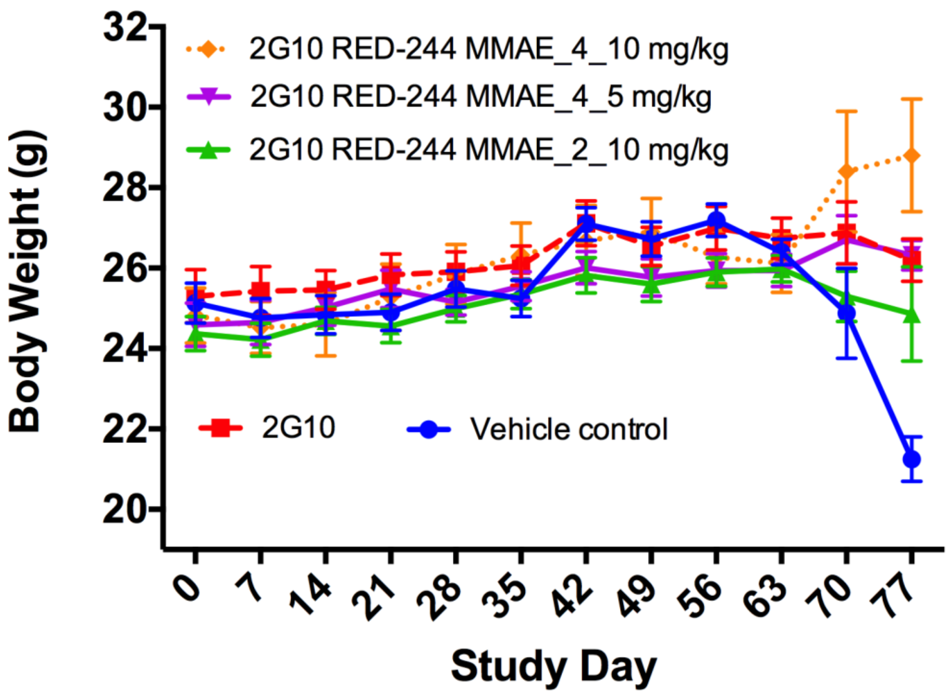

(b)

Figure S1. Mouse body weights with various ADC treatments. Methods for xenograft and ADC treatments are as described in the Methods, section 2.9. Figures A and B are from two different cohorts.
